# Prior Parkinson’s disease diagnosis and subsequent self-reported psychological resilience in the UK Biobank

**DOI:** 10.3389/fpsyg.2026.1906543

**Published:** 2026-09-23

**Authors:** Zhiqiang Xu, Gaiqing Yang, Nan Li

**Affiliations:** 1 Department of Neurology, Zhengzhou Central Hospital Affiliated to Zhengzhou University, Zhengzhou, China; 2 Zhengzhou Key Laboratory of Medical Neurology, Zhengzhou, China; 3 Research Center of Clinical Epidemiology, Peking University Third Hospital, Beijing, China

**Keywords:** brief resilience scale, observational design, Parkinson’s disease, psychological resilience, UK Biobank

## Abstract

**Background:**

The association between psychological resilience and Parkinson’s disease (PD) remains unclear at the population level.

**Methods:**

We analyzed 147,143 UK Biobank participants with available Brief Resilience Scale (BRS) data and complete covariate information. Parkinson’s disease was ascertained using a previously published UK Biobank algorithm. Multivariable logistic regression was used to examine the association between subsequently assessed BRS scores and prior PD after adjustment for demographic, socioeconomic, lifestyle, clinical, and genetic factors. Restricted cubic splines were used to assess nonlinearity.

**Results:**

The mean age was 55.3 years, 56.4% of participants were women, and 346 (0.24%) had Parkinson’s disease. Higher subsequently assessed BRS scores were associated with lower odds of a prior Parkinson’s disease diagnosis. In the fully adjusted model, each 1-point increase in BRS was associated with an OR of 0.63 (95% CI, 0.55–0.73; *p* < 0.001). Compared with the lowest BRS tertile, the OR was 0.63 (95% CI, 0.50–0.80) for the middle tertile and 0.43 (95% CI, 0.31–0.59) for the highest tertile (P for trend<0.001). Inverse associations were also observed for all six BRS items. The overall association was significant in restricted cubic spline analysis (*p* < 0.001), with no evidence of nonlinearity (P for nonlinearity = 0.314). No statistically significant interactions were observed in subgroup analyses. Results were similar in inverse probability-weighted analyses, after additional adjustment for anxiety and depression, and after multiple imputation for missing covariates.

**Conclusion:**

Prior Parkinson’s disease diagnosis was associated with lower subsequently assessed self-reported psychological resilience in the UK Biobank. Given the observational design, causality cannot be established.

## Introduction

Parkinson’s disease (PD) is a chronic and progressive neurodegenerative disorder characterized by motor and non-motor manifestations, and is among the fastest-growing neurological diseases worldwide (Su et al., 2025), with its prevalence having more than doubled over the past two decades (Dorsey et al., 2018; Xu et al., 2026). PD places a substantial burden on patients, caregivers, and healthcare systems (Lu et al., 2024). Despite advances in symptomatic treatment, options for prevention or disease modification remain limited (Tanner and Ostrem, 2024; Winston et al., 2025; Dzamko, 2026). The chronic and progressive nature of PD has also drawn attention to psychological factors related to how individuals respond to and recover from disease-related stress and adversity.

Psychological resilience generally refers to the ability to maintain or regain functioning in the face of stress and adversity (Windle, 2011). It is shaped by a combination of environmental exposures, social context, and genetic factors, reflecting a multidimensional construct (Cornelis et al., 2025). In epidemiologic studies, resilience is often assessed using instruments such as the Brief Resilience Scale (BRS), which captures the capacity to recover from stress (Smith et al., 2008). Psychological resilience has been associated with neuroendocrine and inflammatory responses to stress, although its relevance to Parkinson’s disease remains uncertain (Ménard et al., 2017; Knezevic et al., 2023). In the context of PD, most existing studies have been conducted in clinically diagnosed cohorts, where resilience has been related to depression and demoralization (de Figueiredo et al., 2023), non-motor symptoms (Shamaskin-Garroway et al., 2016), and caregiver mental health (Ertl et al., 2019). However, whether psychological resilience is associated with the presence of PD at the population level remains poorly characterized, particularly in large-scale studies accounting for demographic, lifestyle, clinical, and genetic factors.

To address this gap, we examined the association between prior Parkinson’s disease diagnosis and subsequently assessed self-reported psychological resilience in UK Biobank participants.

## Methods

### Study population

The UK Biobank is a population-based prospective cohort comprising more than 500,000 participants aged 37–73 years who were recruited across the United Kingdom between 2006 and 2010 (Allen et al., 2024). This initial assessment is referred to as the baseline assessment in the present study. At baseline, participants completed touchscreen and interviewer-administered questionnaires, underwent physical measurements, and provided biological samples, with subsequent linkage to health-related records. Subsets of participants were subsequently invited to complete additional follow-up assessments, including online questionnaires. Psychological resilience was assessed as part of a later online mental health questionnaire, as described below.

All participants provided informed consent. The UK Biobank received ethical approval from the North West–Haydock Research Ethics Committee (REC reference: 21/NW/0157) (Sudlow et al., 2015; Rusk, 2018). All study procedures were performed in accordance with the ethical principles of the World Medical Association Declaration of Helsinki.

### Assessment of psychological resilience

Psychological resilience was assessed using the six-item Brief Resilience Scale (BRS) during the UK Biobank follow-up online mental health assessment. The BRS is a validated self-reported measure of perceived ability to recover from stress. The individual items and corresponding UK Biobank field IDs are provided in Supplementary Table S3. Each item is rated on a five-point Likert scale ranging from 1 (“strongly disagree”) to 5 (“strongly agree”). Negatively worded items were reverse-coded according to the standard BRS scoring procedure so that higher scores consistently indicated greater psychological resilience. The overall BRS score was calculated as the mean of the six items, yielding a theoretical range of 1 to 5, consistent with prior UK Biobank studies (Cornelis et al., 2025). The scale demonstrated good internal consistency in the analytical sample (Cronbach’s α = 0.88; 95% CI, 0.87–0.88).

The BRS assessment date was defined as the completion date of the online Social Situation questionnaire containing the BRS items (UK Biobank Field 29,208). For categorical analyses, BRS scores were grouped using tertile-based cutoffs: low (≤3.333), medium (>3.333 to 4.000), and high (>4.000). Because BRS scores take discrete values, participants with identical scores were retained in the same category, resulting in unequal group sizes (Supplementary Table S4).

### Definition of PD

Parkinson’s disease was identified using a previously published UK Biobank algorithm derived from Fields 42,030 and 42,031, which provide the date and source of all-cause parkinsonism reports, respectively (Hughes et al., 2002; Zheng et al., 2023; Geng et al., 2024). Detailed diagnostic definitions, codes, and ascertainment sources are provided in Supplementary Tables S1, S2. Among participants with Parkinson’s disease in the analytical sample, all recorded diagnoses preceded the BRS assessment.

### Covariates

Based on prior literature (Xu et al., 2026; Belete et al., 2023; Peng et al., 2024; Wang et al., 2024), covariates included age, sex, self-reported ethnicity, education, body mass index (BMI), Townsend Deprivation Index (TDI), physical activity, smoking status, alcohol consumption, hypertension, diabetes, stroke, anxiety, depression, and the polygenic risk score for Parkinson’s disease (PD-PRS). Non-genetic covariates were obtained at the UK Biobank baseline assessment conducted between 2006 and 2010. Age was defined at recruitment. Ethnicity was categorized as White or non-White, and education was classified according to educational qualifications. BMI was measured during the baseline assessment, and TDI was assigned according to residential area at recruitment. Physical activity was derived from self-reported moderate- and vigorous-intensity activity. Smoking status was categorized as never, previous, or current, and alcohol consumption as drinker or non-drinker. Hypertension, diabetes, and stroke were defined from baseline self-reports of physician-diagnosed conditions. Self-reported physician-diagnosed anxiety and depression were additionally considered in sensitivity analyses. Detailed UK Biobank field IDs, assessment instances, assessment timing, and coding procedures for all covariates used in the analyses are provided in Supplementary Table S5.

### Genetic susceptibility

PD polygenic risk score was assessed using the UK Biobank Standard Polygenic Risk Score for Parkinson’s disease (Data-Field 26,260), derived from external genome-wide association study data. Genetic ancestry was accounted for within the UK Biobank Standard PRS framework using principal component-based ancestry centering. Details of PRS construction and validation have been reported previously (Hu et al., 2024; Thompson et al., 2024).

### Analytical sample

The BRS assessment occurred several years after the baseline assessment. Participants with available BRS data were considered eligible for the present analysis. Of 502,235 UK Biobank participants, 332,904 were excluded because BRS data were unavailable. Among the remaining 169,331 participants with BRS scores, 22,188 were excluded because of missing data on one or more covariates, including age, sex, ethnicity, education, physical activity, Townsend Deprivation Index, body mass index, smoking status, alcohol consumption, stroke, hypertension, diabetes, or the Parkinson’s disease polygenic risk score. The final analytical sample comprised 147,143 participants (Figure 1). Covariate missingness before complete-case exclusion is summarized in Supplementary Table S6.

**Figure 1:**
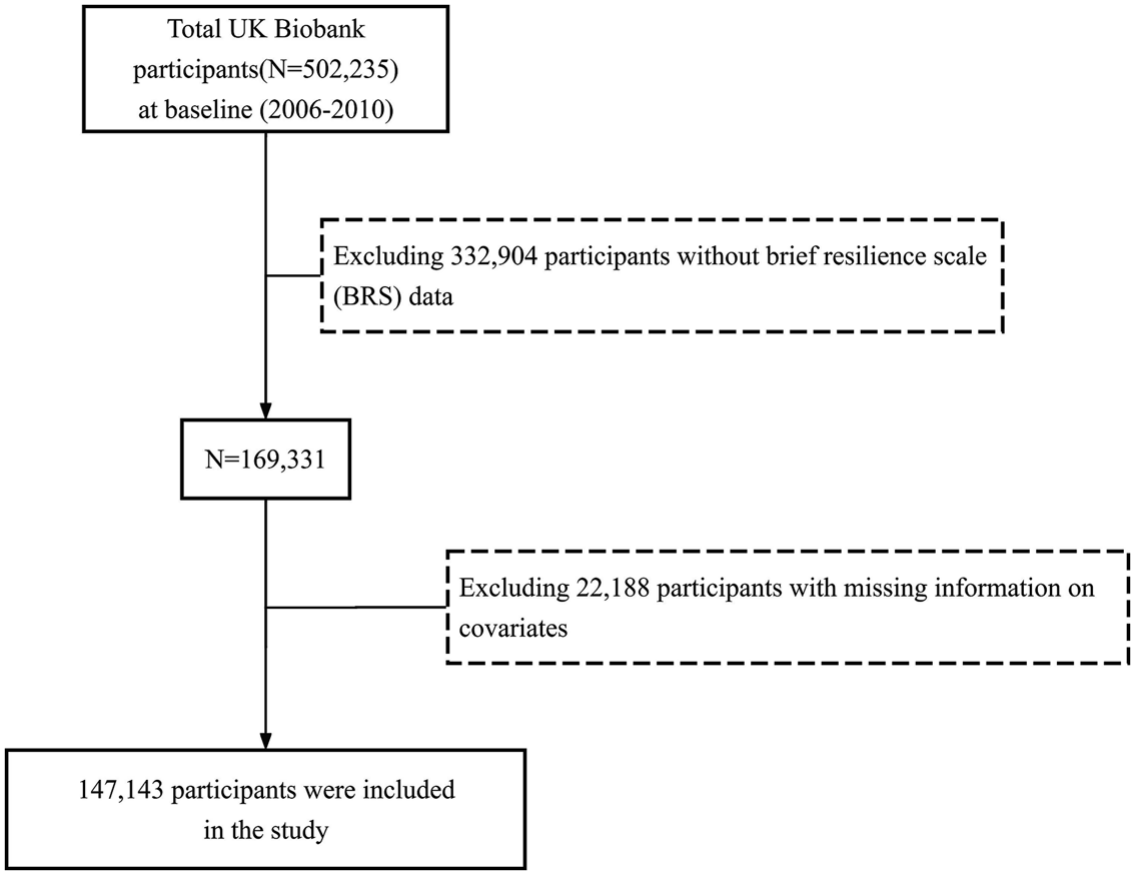
Flowchart of participant inclusion.

### Statistical analysis

Participant characteristics were summarized using means and standard deviations for continuous variables and frequencies and percentages for categorical variables.

Multivariable logistic regression models were used to examine the association between subsequently assessed BRS scores and prior PD. The crude model was unadjusted. Model 1 adjusted for age, sex, and ethnicity. Model 2 further adjusted for education, Townsend Deprivation Index, body mass index, physical activity, smoking status, alcohol consumption, hypertension, diabetes, stroke, and the Parkinson’s disease polygenic risk score.

BRS was analyzed both continuously, with odds ratios (ORs) estimated per 1-point increase, and categorically using tertile-based groups, with the lowest group as the reference. Tests for trend were performed by modeling BRS categories as an ordinal variable. A restricted cubic spline model with three knots placed at the 10th, 50th, and 90th percentiles of the BRS distribution was used to assess potential nonlinearity, with the median BRS score used as the reference.

The six BRS item-level analyses were considered secondary analyses, and *p-*values from the fully adjusted models were adjusted for multiple comparisons using the Benjamini–Hochberg false discovery rate procedure.

Subgroup analyses were conducted according to age, sex, body mass index, education, ethnicity, genetic susceptibility to Parkinson’s disease, physical activity, Townsend Deprivation Index, smoking status, alcohol consumption, hypertension, diabetes, and stroke. Effect modification was assessed by including an interaction term between BRS and each stratification variable in the fully adjusted model. The corresponding stratification variable was omitted from the adjustment set in each subgroup analysis. Subgroup analyses were considered exploratory, and no formal multiplicity adjustment was applied to interaction tests.

Missingness for each variable before complete-case exclusion is reported in Supplementary Table S6. Primary analyses were based on participants with complete data for the variables included in the models; multiple imputation was subsequently performed as a sensitivity analysis to assess the potential influence of missing covariate data.

Sensitivity analyses included inverse probability weighting for potential selection related to BRS availability, additional adjustment for self-reported anxiety and depression, multiple imputation for missing covariates, and replacement of baseline age with age at BRS assessment. Age at BRS assessment was estimated from age at baseline and the interval between the baseline and BRS assessment dates.

All statistical analyses were performed using R version 4.5.1 (R Foundation for Statistical Computing, Vienna, Austria). All tests were two-sided, and *p* < 0.05 was considered statistically significant.

## Results

### Baseline characteristics

Among 502,235 UK Biobank participants, 147,143 were included in the analytical sample. Compared with the full cohort, participants in the analytical sample were slightly younger, more often female and White, had higher educational attainment, and lived in areas with lower socioeconomic deprivation. They also had a lower mean BMI and lower prevalences of current smoking, hypertension, diabetes, stroke, and Parkinson’s disease. The prevalence of Parkinson’s disease was 0.97% in the full cohort and 0.24% in the analytical sample. Characteristics according to BRS participation and analytical sample inclusion are presented in Supplementary Table S7.

Within the analytical sample, participants in higher BRS tertiles were more often male, more likely to meet physical activity recommendations, and less likely to live in areas of high socioeconomic deprivation. The prevalence of Parkinson’s disease was 0.31% in the lowest BRS tertile, 0.22% in the middle tertile, and 0.15% in the highest tertile (Table 1).

**Table 1:** Baseline characteristics of participants across BRS tertiles.

Characteristic	Full UKB cohort	Total	BRS		
			Q1	Q2	Q3
Participants, *N*	502,235	147,143	45,120	68,260	33,763
Age, years	56.53 ± 8.09	55.31 ± 7.54	54.69 ± 7.60	55.69 ± 7.50	55.37 ± 7.50
Sex, %					
Female	273,222 (54.40)	82,992 (56.40)	29,345 (65.04)	37,975 (55.63)	15,672 (46.42)
Male	229,013 (45.60)	64,151 (43.60)	15,775 (34.96)	30,285 (44.37)	18,091 (53.58)
White, %	454,025 (90.90)	135,989 (92.42)	41,630 (92.27)	63,193 (92.58)	31,166 (92.31)
Education, %					
Low	331,070 (67.28)	79,169 (53.80)	24,256 (53.76)	36,365 (53.27)	18,548 (54.94)
High	161,037 (32.72)	67,974 (46.20)	20,864 (46.24)	31,895 (46.73)	15,215 (45.06)
TDI group, %					
Low deprivation	100,086 (19.95)	34,119 (23.19)	10,083 (22.35)	15,962 (23.38)	8,074 (23.91)
Medium deprivation	301,107 (60.03)	91,310 (62.06)	27,802 (61.62)	42,541 (62.32)	20,967 (62.10)
High deprivation	100,416 (20.02)	21,714 (14.76)	7,235 (16.04)	9,757 (14.29)	4,722 (13.99)
BMI, kg/m^2^	27.43 ± 4.80	26.66 ± 4.46	26.51 ± 4.68	26.61 ± 4.36	26.94 ± 4.33
PA, %					
Not meeting	159,750 (37.34)	55,329 (37.60)	18,482 (40.96)	25,297 (37.06)	11,550 (34.21)
Meeting	268,101 (62.66)	91,814 (62.40)	26,638 (59.04)	42,963 (62.94)	22,213 (65.79)
Smoking status, %					
Never	273,386 (54.76)	86,183 (58.57)	26,795 (59.39)	39,682 (58.13)	19,706 (58.37)
Previous	172,955 (34.64)	50,735 (34.48)	15,049 (33.35)	24,037 (35.21)	11,649 (34.50)
Current	52,944 (10.60)	10,225 (6.95)	3,276 (7.26)	4,541 (6.65)	2,408 (7.13)
Alcohol consumption, %	460,124 (91.92)	139,755 (94.98)	42,506 (94.21)	65,097 (95.37)	32,152 (95.23)
Diabetes, %	26,390 (5.28)	4,173 (2.84)	1,391 (3.08)	1850 (2.71)	932 (2.76)
Hypertension, %	135,707 (27.14)	31,314 (21.28)	9,818 (21.76)	14,621 (21.42)	6,875 (20.36)
Stroke, %	7,666 (1.53)	1,066 (0.72)	348 (0.77)	474 (0.69)	244 (0.72)
Depression, %	28,184 (5.61)	7,035 (4.78)	4,643 (10.29)	1927 (2.82)	465 (1.38)
Anxiety, %	6,722 (1.34)	1,679 (1.14)	1,026 (2.27)	522 (0.76)	131 (0.39)
PD, %	4,869 (0.97)	346 (0.24)	141 (0.31)	153 (0.22)	52 (0.15)
PD Genetic risk, %					
Low	162,002 (33.33)	49,048 (33.33)	14,979 (33.20)	22,703 (33.26)	11,366 (33.66)
Medium	162,001 (33.33)	49,047 (33.33)	15,050 (33.36)	22,754 (33.33)	11,243 (33.30)
High	162,001 (33.33)	49,048 (33.33)	15,091 (33.45)	22,803 (33.41)	11,154 (33.04)
BRS	—	3.62 ± 0.74	2.74 ± 0.43	3.74 ± 0.24	4.54 ± 0.31

Continuous variables are presented as mean ± standard deviation (SD), and categorical variables as number (percentage). Percentages were calculated among participants with nonmissing data for the corresponding variable.

UKB, UK Biobank; TDI, Townsend Deprivation Index; BMI, body mass index; PA, physical activity; PD, Parkinson’s disease; BRS, Brief Resilience Scale.

### Association between BRS and Parkinson’s disease

Higher subsequently assessed BRS scores were associated with lower odds of a prior Parkinson’s disease diagnosis. In the fully adjusted model, each 1-point increase in BRS was associated with an OR of 0.63 (95% CI, 0.55–0.73; *p* < 0.001). Compared with participants in the lowest BRS tertile, the OR was 0.63 (95% CI, 0.50–0.80; *p* < 0.001) for the middle tertile and 0.43 (95% CI, 0.31–0.59; *p* < 0.001) for the highest tertile (P for trend<0.001) (Table 2).

**Table 2:** Odds ratios (ORs) and 95% confidence intervals (CIs) for the association between BRS and PD.

Characteristic	Crude model		Model 1		Model 2	
	OR (95% CI)	*p-*value	OR (95% CI)	*p-*value	OR (95% CI)	*p-*value
Per 1-point increase^a^	0.71 (0.62, 0.81)	<0.001	0.62 (0.53, 0.71)	<0.001	0.63 (0.55, 0.73)	<0.001
BRS group^b^						
Low	1 (reference)		1 (reference)		1 (reference)	
Medium	0.72 (0.57, 0.90)	0.004	0.62 (0.49, 0.78)	<0.001	0.63 (0.50, 0.80)	<0.001
High	0.49 (0.36, 0.68)	<0.001	0.41 (0.30, 0.57)	<0.001	0.43 (0.31, 0.59)	<0.001
P for trend		<0.001		<0.001		<0.001

a Odds ratios (ORs) and 95% confidence intervals (CIs) were estimated using logistic regression models. For continuous exposure, the OR represents the odds associated with a per 1-point increase in BRS.

b BRS groups (low, medium, and high) were defined according to tertiles of the Brief Resilience Scale (BRS) distribution in the study population.

The crude model was unadjusted.

Model 1 was adjusted for age, sex, and ethnicity.

Model 2 was further adjusted for education, body mass index (BMI), Townsend Deprivation Index (TDI), physical activity (PA), smoking status, alcohol consumption, hypertension, diabetes, stroke, and Parkinson’s disease polygenic risk score.

OR, odds ratio; CI, confidence interval; BRS, Brief Resilience Scale; PD, Parkinson’s disease.

Similar inverse associations were observed for each of the six BRS items. In the fully adjusted models, the ORs per 1-point increase ranged from 0.74 (95% CI, 0.66–0.83) for Item 3 to 0.80 (95% CI, 0.72–0.88) for Item 6; all associations were statistically significant (all *p* < 0.001) (Table 3). Restricted cubic spline analysis showed a significant overall association between BRS and PD (*p* < 0.001), with no evidence of nonlinearity (P for nonlinearity = 0.314) (Figure 2).

**Table 3:** Association between individual BRS components and PD.

BRS items^a^	Crude model		Model 1		Model 2		FDR-adjusted *p-*value
	OR (95%CI)	*p-*value	OR (95%CI)	*p-*value	OR (95%CI)	*p-*value	
Item 1	0.80 (0.72, 0.89)	<0.001	0.74 (0.66, 0.83)	<0.001	0.75 (0.67, 0.84)	<0.001	<0.001
Item 2	0.81 (0.73, 0.90)	<0.001	0.76 (0.68, 0.85)	<0.001	0.77 (0.69, 0.86)	<0.001	<0.001
Item 3	0.81 (0.72, 0.90)	<0.001	0.74 (0.66, 0.82)	<0.001	0.74 (0.66, 0.83)	<0.001	<0.001
Item 4	0.82 (0.74, 0.92)	<0.001	0.77 (0.69, 0.86)	<0.001	0.78 (0.70, 0.87)	<0.001	<0.001
Item 5	0.79 (0.71, 0.88)	<0.001	0.74 (0.66, 0.82)	<0.001	0.74 (0.67, 0.83)	<0.001	<0.001
Item 6	0.83 (0.75, 0.92)	<0.001	0.79 (0.71, 0.88)	<0.001	0.80 (0.72, 0.88)	<0.001	<0.001

a Items 1–6 represent the six individual items of the Brief Resilience Scale (BRS). ORs and 95% CIs were estimated using logistic regression models, with each item analyzed as a continuous variable (per 1-point increase). *p*-values for the six item-level analyses in the fully adjusted model were adjusted for multiple comparisons using the Benjamini–Hochberg false discovery rate procedure.

The crude model was unadjusted.

Model 1 was adjusted for age, sex, and ethnicity.

Model 2 was further adjusted for education, body mass index (BMI), Townsend Deprivation Index (TDI), physical activity (PA), smoking status, alcohol consumption, hypertension, diabetes, stroke, and Parkinson’s disease polygenic risk score.

OR, odds ratio; CI, confidence interval; BRS, Brief Resilience Scale; PD, Parkinson’s disease.

**Figure 2:**
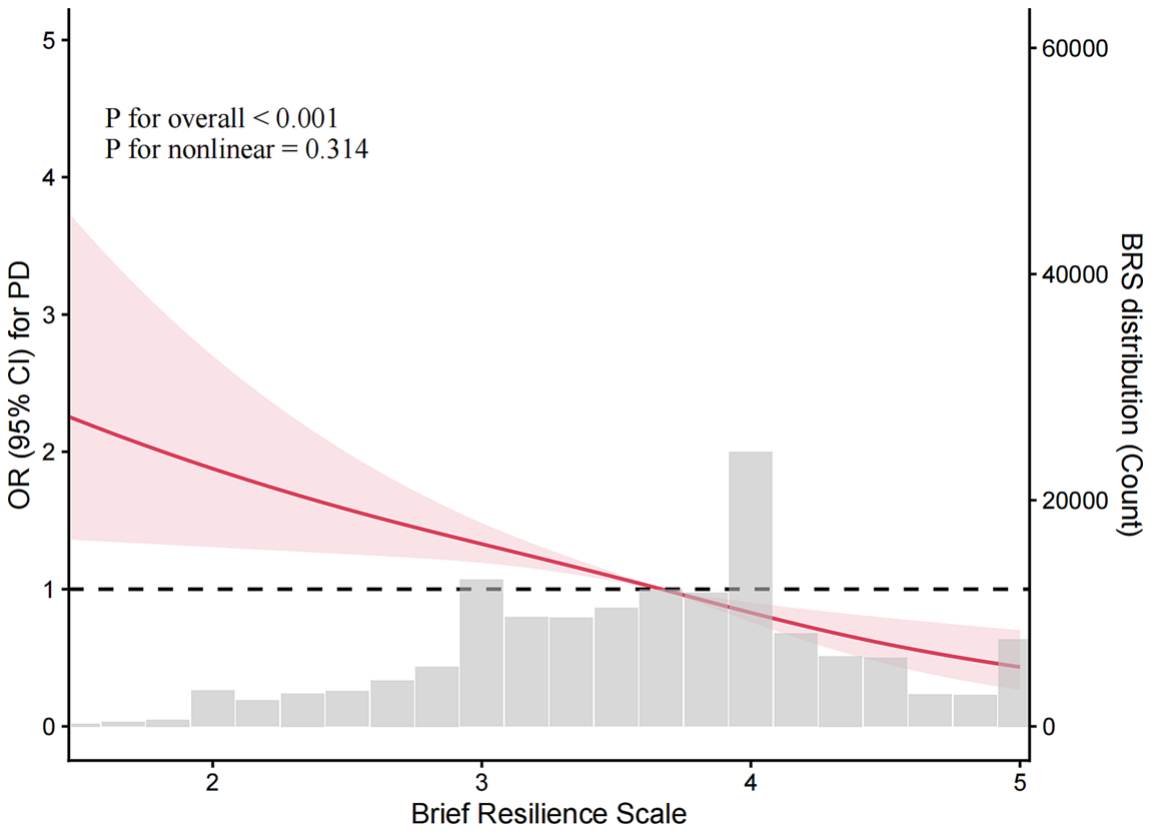
Association between the Brief Resilience Scale and PD. BRS, Brief Resilience Scale; OR, odds ratio; CI, confidence interval; PD, Parkinson’s disease. Adjusted for age, sex, ethnicity, education, Townsend Deprivation Index group, drinking status, smoking status, body mass index, physical activity, hypertension, diabetes, stroke, and Parkinson’s disease polygenic risk score.

### Additional analyses

Exploratory subgroup analyses are presented in Supplementary Figure S1. The association between BRS and prior PD was examined across subgroups defined by age, sex, BMI, education level, ethnicity, genetic risk, physical activity, socioeconomic status (TDI), smoking status, alcohol consumption, diabetes, hypertension, and stroke. No statistically significant interactions were observed for these variables (all P for interaction > 0.05). Given the exploratory nature of these analyses, the estimates were interpreted with attention to their 95% confidence intervals and precision.

Sensitivity analyses yielded similar estimates. The fully adjusted OR per 1-point increase in BRS was 0.63 (95% CI, 0.55–0.73) when age at baseline was replaced with age at BRS assessment, 0.63 (95% CI, 0.55–0.74) in the inverse probability-weighted analysis, 0.66 (95% CI, 0.57–0.77) after additional adjustment for self-reported anxiety and depression, and 0.64 (95% CI, 0.56–0.73) after multiple imputation for missing covariates (Supplementary Tables S8–S11).

## Discussion

In this observational analysis of 147,143 UK Biobank participants, prior Parkinson’s disease diagnosis was associated with lower subsequently assessed self-reported psychological resilience. The association was evident across continuous and categorical BRS analyses and remained similar in sensitivity analyses. To our knowledge, few large population-based studies (Erro et al., 2022; van der Heide et al., 2020; Pauly et al., 2022) have examined the relationship between psychological resilience and PD. Our findings extend previous clinical observations by demonstrating an association between prior Parkinson’s disease diagnosis and subsequently assessed self-reported resilience in a large UK Biobank sample.

Previous studies of psychological resilience in Parkinson’s disease have largely focused on patients with established disease, particularly in the context of the COVID-19 pandemic. Erro et al. (2022) reported that lower resilience was associated with greater psychological distress and poorer health-related quality of life among patients with Parkinson’s disease. Similarly, van der Heide et al. (2020) found that greater resilience was associated with lower perceived stress during the pandemic. Pauly et al. (2022) further showed that many patients with Parkinson’s disease maintained normal or high resilience despite pandemic-related stress, although depressive symptoms were increased in the pandemic-exposed group. These studies suggest that resilience is relevant to psychological adaptation after Parkinson’s disease has developed. Our findings extend this literature by showing that participants with a prior Parkinson’s disease diagnosis reported lower subsequent BRS scores in a large population-based sample. Given the observational design, the association does not establish causality, and lower subsequently reported resilience may partly reflect psychological and functional consequences of established Parkinson’s disease.

Resilience appears to capture dimensions of disease experience that are not fully explained by motor pathology. Prior clinical studies have shown that greater resilience correlates strongly with disability and quality of life but only weakly with motor severity (Shamaskin-Garroway et al., 2016; Robottom et al., 2012), suggesting that resilience reflects adaptive capacity rather than dopaminergic motor function per se. Resilience has also been shown to align more closely with demoralization than with depressive symptoms alone (de Figueiredo et al., 2023), suggesting that it captures a related but distinct aspect of the psychosocial profile in PD. These observations suggest that resilience may reflect aspects of psychosocial adaptation among individuals with Parkinson’s disease.

Higher subsequently assessed BRS scores were associated with lower odds of a prior Parkinson’s disease diagnosis. One possible interpretation is that perceived psychological resilience may reflect differences in how individuals respond and adapt to chronic stress after Parkinson’s disease has developed. Previous studies have linked stress-related trajectories in Parkinson’s disease to psychosocial resources and adaptation (van der Heide et al., 2024). Although neuroendocrine and inflammatory responses to stress may be relevant to the broader experience of chronic disease (O'Callaghan and Miller, 2019; Soares et al., 2019), these pathways were not assessed in the present study and should not be interpreted as mechanisms underlying the observed association.

The BRS captures a self-reported psychosocial construct reflecting perceived ability to recover from stress. It should therefore be distinguished from biological or neurological concepts of resilience, which were not assessed in this study. The observed association is best interpreted in relation to perceived psychological resilience rather than underlying neurobiological reserve or resistance to neuropathology.

### Strengths and limitations

This study has several strengths. It included a large overall sample, a broad range of covariates, and several sensitivity analyses. Resilience was assessed using a validated scale, and results were consistent across individual items. Sensitivity analyses yielded estimates similar to the primary analysis, and subgroup analyses showed no statistically significant evidence of effect modification.

Several limitations should be considered. First, although Parkinson’s disease diagnoses preceded BRS assessment, the observational design does not establish causality. Because the BRS was assessed during a later follow-up assessment and the Parkinson’s disease diagnoses included in the present analysis occurred before BRS assessment, this study could not evaluate whether psychological resilience predicts subsequent incident Parkinson’s disease. Reverse causation remains possible because psychological, cognitive, motor, and functional changes associated with established Parkinson’s disease may influence subsequent BRS responses. BRS responses may also partly reflect participants’ current psychological, cognitive, and functional status, particularly among those with established Parkinson’s disease. Second, resilience was assessed at a single time point and may not capture changes over time. Third, most non-genetic covariates were measured at the UK Biobank baseline assessment, whereas BRS was assessed later; changes in lifestyle or clinical status during this interval may therefore have resulted in residual confounding. Fourth, BRS data were available only for participants who completed the follow-up online mental health assessment. Participants with advanced Parkinson’s disease, cognitive impairment, substantial disability, institutionalization, or limited digital access may have been less likely to complete the questionnaire. Selection related to healthy-volunteer effects, survival, digital participation, and complete-case inclusion therefore cannot be excluded, although the findings were similar in inverse probability-weighted analyses. Fifth, residual and unmeasured confounding cannot be excluded despite adjustment for a broad range of sociodemographic, lifestyle, clinical, and genetic factors. Although self-reported anxiety and depression were additionally considered in sensitivity analyses, other potentially relevant factors, including perceived stress, cognitive impairment, Parkinson’s disease duration, motor and nonmotor symptom burden, functional disability, medication use, and social support, were not available or were not included in the present analysis. The findings should therefore be generalized cautiously, particularly to populations with greater disease severity, functional impairment, limited digital access, or more diverse ancestral backgrounds. Future longitudinal studies with resilience assessed before Parkinson’s disease onset and repeated over time are needed to clarify the temporal relationship.

## Conclusion

In this large UK Biobank study, prior Parkinson’s disease diagnosis was associated with lower subsequently assessed self-reported psychological resilience. The association was similar in sensitivity analyses, and no statistically significant effect modification was identified in exploratory subgroup analyses. Given the observational design, causality cannot be established, and disease-related influences on subsequent BRS responses remain possible.

## Data Availability

The study was conducted under the UK Biobank project application number 106707. Requests to access these datasets should be directed to the UK Biobank team (https://www.ukbiobank.ac.uk/). Any additional inquiries can be directed to the corresponding authors.
