# Supplementary material for: Prior Parkinson’s disease diagnosis and subsequent self-reported psychological resilience in the UK Biobank
Source: Front Psychol. 2026 Sep 23;17:1906543. doi: 10.3389/fpsyg.2026.1906543 (PMC13642686; doi:10.3389/fpsyg.2026.1906543)
Supplement: Supplementary file 1 [file Supplementary_file_1.docx]

**Table S1.** Ascertainment of Parkinson’s disease cases in the UK Biobank

| **ICD-9** | **ICD-10** | **Self-reported fields** |
| --- | --- | --- |
| 332.0,  332.1,  333.0 | G20, G21, G21.0, G21.1, G21.2, G21.3, G21.4, G21.8, G21.9, G22, G23.0, G23.1, G23.2, G23.3, G23.8, G23.9, G25.9, G26, G90.3 | 20002(1262) |

Parkinson’s disease was ascertained using International Classification of Diseases, Ninth and Tenth Revision (ICD-9 and ICD-10) codes and self-reported diagnoses according to a previously published UK Biobank definition.^[1]^
****Abbreviations:**** ICD, International Classification of Diseases.

**Table S2.** Sources of Parkinson’s disease Ascertainment Among Cases in the Analytical Sample

| **Source** | **PD cases, n** | **%** |
| --- | --- | --- |
| Self-reported only | 42 | 12.14 |
| Hospital record, primary diagnosis | 24 | 6.94 |
| Hospital record, secondary diagnosis | 280 | 80.92 |
| Total | 346 | 100.00 |

Source categories were derived from UK Biobank Field 42031 and the corresponding UK Biobank outcome source coding.

**Table S3.** UK Biobank field IDs and item descriptions for the Brief Resilience Scale (BRS)

| **Label** | **BRS item**  **How much do you agree with the following (strongly disagree, disagree, neutral, agree, strongly agree)** | **UKB Field** |
| --- | --- | --- |
| **Item1**  **Item2**  **Item3**  **Item4**  **Item5**  **Item6** | I tend to take a long time to get over setbacks in my life (reverse scoring)  It is hard for me to snap back when something bad happens (reverse scoring)  I usually come through difficult times with little trouble  I have a hard time making it through stressful events (reverse scoring)  It does not take me long to recover from a stressful event  I tend to bounce back quickly after hard times | 29180  29178  29179  29176  29177  29175 |

### Note. The items listed are from the Brief Resilience Scale (BRS), a 6-item self-report measure assessing an individual’s ability to bounce back from stress. Each item is rated on a 5-point Likert scale ranging from strongly disagree (1) to strongly agree (5). Responses were recoded to the conventional 1–5 BRS metric. Negatively worded items were reverse-scored, and the overall BRS score was calculated as the mean of the six items, ranging from 1 to 5.^[2]^

**Abbreviations:** BRS, Brief Resilience Scale.

**Table S4.** Cut-off Values and Distribution of Brief Resilience Scale Tertiles

| **BRS tertile** | **BRS range** | **N** | **%** |
| --- | --- | --- | --- |
| Low (Q1) | 1.000–3.333 | 45,120 | 30.66 |
| Middle (Q2) | >3.333–4.000 | 68,260 | 46.39 |
| High (Q3) | >4.000–5.000 | 33,763 | 22.95 |

BRS, Brief Resilience Scale. Tertiles were defined according to the observed distribution of the BRS score. Because the BRS score takes discrete values, participants with identical scores were retained in the same category, resulting in unequal group sizes.

**Table S5.** UK Biobank Field IDs, Assessment Timing, and Coding of Covariates Used in the Analyses

| **Variable** | **Field ID** | **Question or description** | **Categories from raw data** | **Categories for the current study** |
| --- | --- | --- | --- | --- |
| Age | 21022 | Age at recruitment | Continuous, years. | Continuous in the primary models; categorized as <65 and ≥65 years for subgroup analyses. |
| Sex | 31 | Sex | 0 = Female; 1 = Male. | Female; Male. |
| Ethnicity | 21000 | Ethnic background | White; Mixed; Asian or Asian British; Black or Black British; Chinese; Other ethnic group | White = codes 1, 1001, 1002, 1003; Non-White = all other valid ethnic categories; −1, −3, and missing values were treated as missing |
| Education | 6138 | Qualifications | College or University degree;  A levels/AS levels or equivalent;  O levels/GCSEs or equivalent;  CSEs or equivalent;  NVQ or HND or HNC or  equivalent;  Other professional qualifications eg: nursing, teaching. | High level = College or university degree;  Low level = Below college or university degree. |
| Townsend deprivation index gruop | 22189 | Townsend deprivation index at  recruitment | Continuous. | Based on the distribution of TDI in the UK Biobank baseline population, participants were categorized into tertiles:Low deprivation: TDI < –3.93; Medium deprivation: –3.93 ≤ TDI < 1.35; High deprivation: TDI ≥ 1.35. |
| Smoking status | 20116 | The current/past smoking status of the participant | Never;  Past;  Current. | Never = 0;  Previous = 1;  Current = 2. |
| Drinking | 20117 | Alcohol drinker status | Never;  Previous;  Current. | No=0 or 1;  Yes=2. |
| Physical activity | 884,894,  904,914 | Number of days/week of  moderate physical activity 10+ minutes;  Duration of moderate activity;  Number of days/week of  vigorous physical activity 10+ minutes;  Duration of vigorous activity. | Continuous, minutes/day;  days/week. | 1 = < 150 minutes moderate activity per week OR < 75 minutes vigorous activity per week;  0 = ≥150 minutes moderate activity per week OR ≥ 75 minutes vigorous activity per week OR equivalent combination. |
| Body mass index | 21001 | Body mass index (BMI) | kg/m^2^. | Continuous. |
| Hypertension | 6150 | Vascular/heart problems diagnosed by doctor | High blood pressure. | No=1 , 2,3,-7;  Yes=4. |
| Diabetes | 2443 | Diabetes diagnosed by doctor | No;  Yes. | No=0;  Yes=1. |
| Stroke | 6150 | Vascular/heart problems diagnosed by doctor | Stroke. | Stroke = code 3 recorded in any array position; non-stroke = no code 3 among valid responses. |
| PD-PRS | 26260 | Standard PRS for parkinson's disease (PD) | Continuous. | low (≤−0.578012),  medium (>−0.578012 to ≤0.293616),  high (>0.293616). |

Unless otherwise indicated, non-genetic covariates were obtained from assessment instance 0 at the UK Biobank baseline assessment conducted between 2006 and 2010.

**Table S6.** Missingness of Study Variables Before Complete-Case Analysis

| **Variable** | **Missing, n** | **Missing, %** |
| --- | --- | --- |
| Age | 0 | 0 |
| Sex | 0 | 0 |
| Ethnicity | 602 | 0.36 |
| Education | 1,703 | 1.01 |
| Townsend Deprivation Index | 190 | 0.11 |
| Body mass index | 397 | 0.23 |
| Physical activity | 17,164 | 10.14 |
| Smoking status | 405 | 0.24 |
| Alcohol consumption | 154 | 0.09 |
| Hypertension | 272 | 0.16 |
| Diabetes | 314 | 0.19 |
| Stroke | 272 | 0.16 |
| Parkinson’s disease polygenic risk score | 4,382 | 2.59 |

Missingness was assessed among 169,331 participants with available Brief Resilience Scale data before exclusion for incomplete covariate information. Percentages were calculated using 169,331 as the denominator.

**Table S7.** Characteristics of Participants According to BRS Participation and Inclusion in the Analytical Sample

| **Characteristic** | **Full UKB cohort** | **BRS nonrespondents** | **BRS respondents excluded for missing covariates** | **Analytical sample** |
| --- | --- | --- | --- | --- |
| Participants, n | 502,235 | 332,904 | 22,188 | 147,143 |
| Age, years | 56.53 ± 8.09 | 57.10 ± 8.31 | 56.15 ± 7.37 | 55.31 ± 7.54 |
| Female, n (%) | 273,222 (54.40) | 175,533 (52.73) | 14,697 (66.24) | 82,992 (56.40) |
| White ethnicity, n (%) | 454,025 (90.90) | 298,145 (90.15) | 19,891 (92.15) | 135,989 (92.42) |
| High education, n (%) | 161,037 (32.72) | 85,701 (26.41) | 7,362 (35.94) | 67,974 (46.20) |
| High deprivation, n (%) | 100,416 (20.02) | 74,924 (22.54) | 3,778 (17.17) | 21,714 (14.76) |
| BMI, kg/m² | 27.43 ± 4.80 | 27.79 ± 4.91 | 27.19 ± 4.84 | 26.66 ± 4.46 |
| Current smoking, n (%) | 52,944 (10.60) | 41,082 (12.44) | 1,637 (7.52) | 10,225 (6.95) |
| Hypertension, n (%) | 135,707 (27.14) | 99,033 (29.92) | 5,360 (24.46) | 31,314 (21.28) |
| Diabetes, n (%) | 26,390 (5.28) | 21,482 (6.50) | 735 (3.36) | 4,173 (2.84) |
| Stroke, n (%) | 7,666 (1.53) | 6,410 (1.94) | 190 (0.87) | 1,066 (0.72) |
| PD, n (%) | 4,869 (0.97) | 4,457 (1.34) | 66 (0.30) | 346 (0.24) |

Continuous variables are presented as mean ± standard deviation, and categorical variables as number (percentage). The table is descriptive and intended to characterize differences in participant selection across stages of the analytical sample construction.

**Table S8.** Sensitivity Analysis of the Association Between Brief Resilience Scale Scores and Parkinson’s Disease Using Age at BRS Assessment

| **Characteristic** | **Crude model** | |  | **Model 1** | |  | **Model 2** | |
| --- | --- | --- | --- | --- | --- | --- | --- | --- |
|  | **OR(95% CI)** | ***p* Value** |  | **OR(95% CI)** | ***p* Value** |  | **OR(95% CI)** | ***p* Value** |
| **Per 1-point increase^a^** | 0.71(0.62,0.81) | <0.001 |  | 0.62(0.54,0.72) | <0.001 |  | 0.63(0.55,0.73) | <0.001 |
| **BRS group^b^** |  |  |  |  |  |  |  |  |
| Low | 1(reference) |  |  | 1(reference) |  |  | 1(reference) |  |
| Medium | 0.72(0.57,0.90) | 0.028 |  | 0.62(0.49–0.78) | 0.001 |  | 0.63(0.50–0.80) | <0.001 |
| High | 0.49(0.36,0.68) | <0.001 |  | 0.42(0.30–0.57) | <0.001 |  | 0.43(0.31–0.59) | <0.001 |
| **P for trend** |  | <0.001 |  |  | <0.001 |  |  | <0.001 |

^a^Odds ratios (ORs) and 95% confidence intervals (CIs) were estimated using logistic regression. BRS was analyzed continuously, with the OR expressed per 1-point increase in the mean BRS score.

**^b^**BRS groups (low, medium, and high) were defined according to tertiles of the Brief Resilience Scale (BRS) distribution in the study population.

The crude model was unadjusted.

Model 1 was adjusted for age at BRS assessment, sex, and ethnicity.

Model 2 was further adjusted for education, body mass index (BMI), Townsend Deprivation Index (TDI), physical activity (PA), smoking status, alcohol consumption, hypertension, diabetes, stroke, and Parkinson’s disease polygenic risk score.

Age at BRS assessment was estimated from age at baseline and the interval between the baseline assessment date and BRS assessment date.

**Abbreviations:** OR, odds ratio; CI, confidence interval; IPW, inverse probability weighting; BRS, Brief Resilience Scale; PD, Parkinson’s disease.

**Table S9.** Inverse Probability-Weighted Associations of the Brief Resilience Scale With Parkinson’s disease

| **Characteristic** | **Crude model** | |  | **Model 1** | |  | **Model 2** | |
| --- | --- | --- | --- | --- | --- | --- | --- | --- |
|  | **OR(95% CI)** | ***p* Value** |  | **OR(95% CI)** | ***p* Value** |  | **OR(95% CI)** | ***p* Value** |
| **Per 1-point increase^a^** | 0.71(0.62–0.81) | <0.001 |  | 0.62(0.53,0.72) | <0.001 |  | 0.63(0.55,0.74) | <0.001 |
| **BRS group^b^** |  |  |  |  |  |  |  |  |
| Low | 1(reference) |  |  | 1(reference) |  |  | 1(reference) |  |
| Medium | 0.76(0.60–0.97) | 0.028 |  | 0.67(0.52–0.85) | 0.001 |  | 0.68(0.53–0.87) | 0.002 |
| High | 0.49(0.35–0.69) | <0.001 |  | 0.42(0.30–0.59) | <0.001 |  | 0.43(0.31–0.61) | <0.001 |
| **P for trend** |  | <0.001 |  |  | <0.001 |  |  | <0.001 |

^a^Odds ratios (ORs) and 95% confidence intervals (CIs) were estimated using inverse probability-weighted logistic regression models with robust standard errors. Stabilized inverse probability weights for participation in the BRS assessment were truncated at the 1st and 99th percentiles. For continuous BRS, ORs represent the odds ratio for PD per 1-point increase in BRS.

**^b^**BRS groups (low, medium, and high) were defined according to tertiles of the Brief Resilience Scale (BRS) distribution in the study population.

The crude model was unadjusted.

Model 1 was adjusted for age, sex, and ethnicity.

Model 2 was further adjusted for education, body mass index (BMI), Townsend Deprivation Index (TDI), physical activity (PA), smoking status, alcohol consumption, hypertension, diabetes, stroke, and Parkinson’s disease polygenic risk score.

**Abbreviations:** OR, odds ratio; CI, confidence interval; IPW, inverse probability weighting; BRS, Brief Resilience Scale; PD, Parkinson’s disease.

**Table S10.** Association Between the Brief Resilience Scale and Parkinson’s disease After Additional Adjustment for Anxiety and Depression

| **Characteristic** |  | **OR(95% CI)** | ***p* Value** |
| --- | --- | --- | --- |
| **Per 1-point increase^a^** |  | 0.66(0.57,0.77) | <0.001 |
| **BRS group^b^** |  |  |  |
| Low |  | 1(reference) |  |
| Medium |  | 0.67(0.53,0.85) | <0.001 |
| High |  | 0.46(0.33,0.63) | <0.001 |
| **P for trend** |  |  | <0.001 |

**^a^**Odds ratios (ORs) and 95% confidence intervals (CIs) were estimated using logistic regression models. For continuous exposure, the OR represents the odds associated with a per 1-point increase in BRS.

**^b^**BRS groups (low, medium, and high) were defined according to tertiles of the Brief Resilience Scale (BRS) distribution in the study population.

The model was adjusted for age, sex, ethnicity, education, body mass index (BMI), Townsend Deprivation Index (TDI), physical activity (PA), smoking status, alcohol consumption, hypertension, diabetes, stroke, the Parkinson’s disease polygenic risk score, and additionally for self-reported anxiety and depression.

**Abbreviations:** OR, odds ratio; CI, confidence interval; BRS, Brief Resilience Scale; PD, Parkinson’s disease.

**Table S11.** Associations of the Brief Resilience Scale With Parkinson’s disease After Multiple Imputation

| **Characteristic** | **Crude model** | |  | **Model 1** | |  | **Model 2** | |
| --- | --- | --- | --- | --- | --- | --- | --- | --- |
|  | **OR(95% CI)** | ***p* Value** |  | **OR(95% CI)** | ***p* Value** |  | **OR(95% CI)** | ***p* Value** |
| **Per 1-point increase^a^** | 0.71(0.63,0.81) | <0.001 |  | 0.62(0.55,0.71) | <0.001 |  | 0.64 (0.56–0.73) | <0.001 |
| **BRS group^b^** |  |  |  |  |  |  |  |  |
| Low | 1(reference) |  |  | 1(reference) |  |  | 1(reference) |  |
| Medium | 0.71(0.57,0.87) | 0.001 |  | 0.62(0.50,0.76) | <0.001 |  | 0.63 (0.51–0.78) | <0.001 |
| High | 0.49(0.36,0.65) | <0.001 |  | 0.42 (0.31–0.56) | <0.001 |  | 0.43 (0.32–0.58) | <0.001 |
| **P for trend** |  | <0.001 |  |  | <0.001 |  |  | <0.001 |

**^a^**Odds ratios (ORs) and 95% confidence intervals (CIs) were estimated using logistic regression models. For continuous exposure, the OR represents the odds associated with a per 1-point increase in BRS.

**^b^**BRS groups (low, medium, and high) were defined according to tertiles of the Brief Resilience Scale (BRS) distribution in the study population.

The crude model was unadjusted.

Model 1 was adjusted for age, sex, and ethnicity.

Model 2 was further adjusted for education, body mass index (BMI), Townsend Deprivation Index (TDI), physical activity (PA), smoking status, alcohol consumption, hypertension, diabetes, stroke, and Parkinson’s disease polygenic risk score.

**Abbreviations:** OR, odds ratio; CI, confidence interval; BRS, Brief Resilience Scale; PD, Parkinson’s disease.

**Figure S1.** Association Between the Brief Resilience Scale and Parkinson’s Disease Across Subgroups

**Abbreviations:** OR, odds ratio; CI, Confidence Interval; BMI, body mass index; PD, Parkinson’s disease.

Adjusted for age group, sex, ethnicity, education, townsend deprivation index group, drinking status, smoking status, body mass index group, physical activity, hypertension, diabetes, stroke, and PD polygenic risk score.

**References**

[1]	Thompson DJ, Wells D, Selzam S, et al. A systematic evaluation of the performance and properties of the UK Biobank Polygenic Risk Score (PRS) Release. PLoS One. 2024. 19(9): e0307270.

[2]	Cornelis MC, Caldwell JA, Vu T, Hennigar SR, Berryman CE, Lieberman HR. The brief resilience scale: a genome-wide association study in the UK Biobank. BMC Med. 2025. 23(1): 520.
